# Supplementary material for: Temporal modulation of the NF-κB RelA network in response to different types of DNA damage
Source: Biochem J. 2021 Feb 10;478(3):533–51. doi: 10.1042/BCJ20200627 (PMC7886319; doi:10.1042/BCJ20200627)
Supplement: Supplementary Figures S1-S3 [file BCJ-478-533-s1.pdf]

Supplementary Table Legends

Supplementary Table 1. Kmeans clustering of phosphoproteins in the RelA interactome network. RelA associated proteins were inputted into STRING and clustered using the Kmenas algorithm. Proteins associated with each of the 9 clusters are listed along with the categories as depicted in Figure 2.

Supplementary Table 2. Temporal phosphopeptide response in the RelA network to cellular treatment with etoposide (ETO). List of peptides (and the proteins from which they derived) identified by LC-MS/MS in response to cellular exposure of HA-RelA U2OS cells to 50 µM ETO for 30 min, 60 min or 120 min (or control treatment with DMSO), followed by HA-RelA immunoprecipitation and TiO2-based phosphopeptide enrichment. Peptide identification metrics, protein modifications, as well as the confidence of phosphosite localisation (ptmRS score) are detailed for each entry. Peptide quantification was performed following VSN normalisation of peptide ion abundances. ANOVA was performed to evaluate statistical changes across all time points. Paired t-test was used to compare two individual time points. Confidently localised phosphorylation sites were evaluated using NetPhorest to predict kinase family (group) and kinase. Also detailed is the prevalence of prior observation of the identified phosphopeptides in either PhosphositePlus (PSP) or Peptide Atlas (PA) – see Methods for detailed information.

Supplementary Table 3. Temporal phosphopeptide response in the RelA network to cellular treatment with hydroxyurea (HU). List of peptides (and the proteins from which they derived) identified by LC-MS/MS in response to cellular exposure of HA-RelA U2OS cells to 2 mM HU for 30 min, 60 min or 120 min (or control treatment with DMSO), followed by HA-RelA immunoprecipitation and TiO2-based phosphopeptide enrichment. Peptide identification metrics, protein modifications, as well as the confidence of phosphosite localisation (ptmRS score) are detailed for each entry. Peptide quantification was performed following VSN normalisation of peptide ion abundances. ANOVA was performed to evaluate statistical changes across all time points. Paired t-test was used to compare two individual time points. Confidently localised phosphorylation sites were evaluated using NetPhorest to predict kinase family (group) and kinase. Also detailed is the prevalence of prior observation of the identified phosphopeptides in either PhosphositePlus (PSP) or Peptide Atlas (PA) – see Methods for detailed information.

Supplementary Figure Legends

Supplementary Figure 1. Time course of treatment of HA-RelA U2OS cells with etoposide or hydroxyurea. HA-RelA U2OS cells were treated for the specified times with either etoposide (A) or hydroxyurea (B) as indicated to assess DNA damage. Cell lysates were separate by SDS-PAGE; total protein was visualised with Ponceau S staining. Gels were then subjected to immunoblotting with antibodies against either γH2AX as a marker of DNA damage, or RelA/p65. MM: molecular markers; WCL: whole cell lysate; CL: cleared lysate; Elution: eluant from anti-HA beads; CNT: control. Control cells for hydroxyurea treatment were exposed to DMSO for 6 h to match longest treatment time.

Supplementary Figure 2 GIFs. Animated dynamic network map of RelA interacting phosphoproteins. Significant changes across either (A) etoposide and/or hydroxyurea, or (B) both treatments as a function of time (using data presented in Fig. 2).

Supplementary Figure 3. Schematic of RelA protein coverage. Domains and quantified sites of phosphorylation are indicated. Blue phosphorylation sites were quantified in ETO only, red were quantified in both ETO and HU. Novel phosphorylation sites are represented with an asterix. Protein sequence coverage is represented by the boxes (and amino acid residue numbers) in the bottom panel. TA = transactivation domain; NLS – nuclear localisation sequence*.*

A

Etoposide stimulation

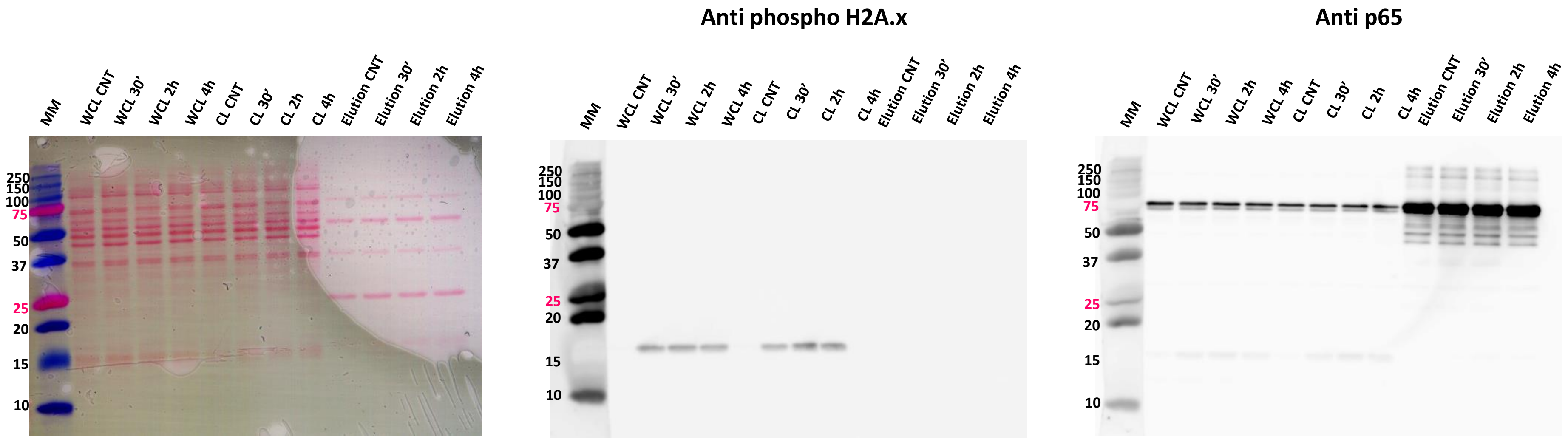

B

Hydroxyurea stimulation

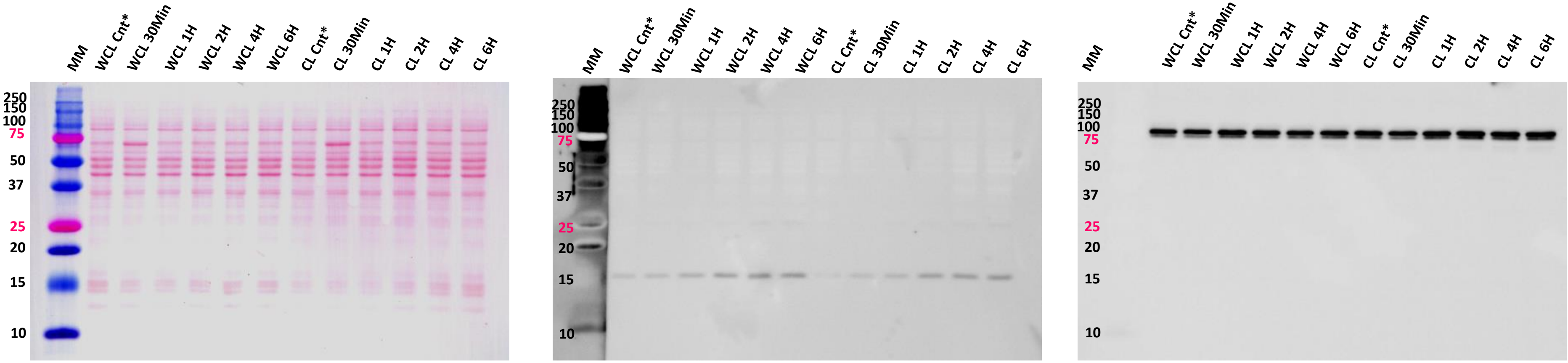

WCL – whole cell lysate
CL – cleared lysate
Elution – Elution of the Anti-HA beads
*** In HU control cells were exposed to 6h of DMSO**

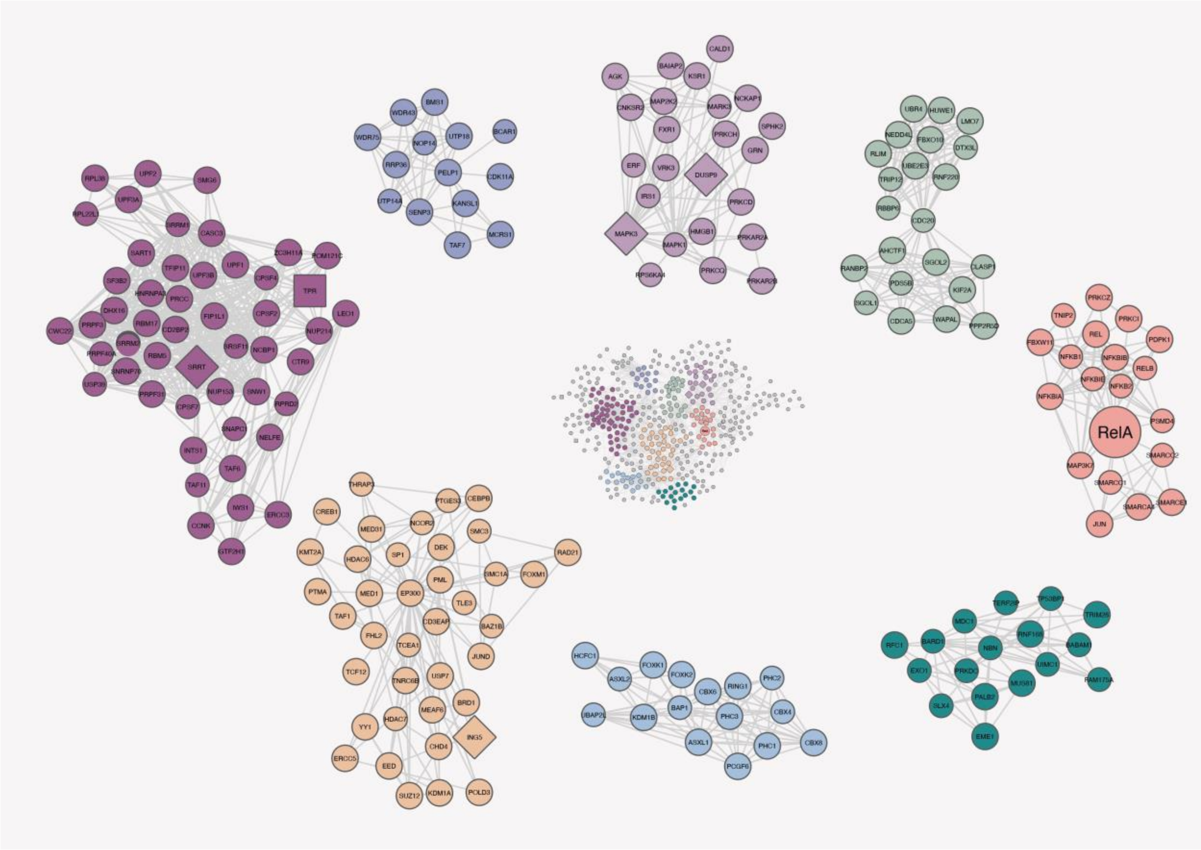

RPL38
UPF2
SMG6
UPF3A
RPL22L1
SRRM1
CASC3
SART1
TFIP11
UPF3B
UPF1
ZC3H11A
POM121C
SF3B2
HNRNPA3
PRCC
CPSF4
TPR
DHX16
FIP1L1
CPSF2
LEO1
CWC22
PRPF3
RBM17
CD2BP2
NUP214
SRRM2
PRPF40A
RBM5
SRSF11
NCBP1
SRRT
CTR9
SNRNP70
USP39
PRPF31
CPSF7
NUP153
SNW1
RPRD2
SNAPC1
NELFE
INTS1
TAF6
TAF11
IWS1
ERCC3
CCNK
GTF2H1
BMS1
WDR43
WDR75
NOP14
UTP18
BCAR1
RRP36
PELP1
CDK11A
UTP14A
SENP3
KANSL1
MCRS1
TAF7
CALD1
BAIAP2
AGK
KSR1
CNKSR2
MAP2K2
MARK3
NCKAP1
SPHK2
FXR1
PRKCH
GRN
ERF
VRK3
DUSP9
IRS1
PRKCD
MAPK3
MAPK1
HMGB1
PRKAR2A
RPS6KA4
PRKCQ
PRKAR2B
UBR4
HUWE1
LMO7
NEDD4L
FBXO10
DTX3L
RLIM
UBE2E3
TRIP12
RNF220
RBBP6
CDC20
AHCTF1
SGOL2
RANBP2
CLASP1
PDS5B
KIF2A
SGOL1
CDCA5
WAPAL
PPP2R5D
PRKCZ
TNIP2
PRKCI
REL
FBXW11
PDPK1
NFKB1
NFKBIB
RELB
NFKBIE
NFKB2
NFKBIA
RelA
PSMD4
SMARCC2
MAP3K7
SMARCC1
SMARCA4
SMARCE1
JUN
THRAP3
PTGES3
CEBPB
CREB1
MED31
NCOR2
SMC3
KMT2A
HDAC6
SP1
DEK
RAD21
PML
SMC1A
FOXM1
PTMA
MED1
EP300
TLE3
TAF1
CD3EAP
BAZ1B
FHL2
TCEA1
JUND
TCF12
TNRC6B
USP7
BRD1
MEAF6
HDAC7
YY1
ING5
CHD4
ERCC5
EED
SUZ12
KDM1A
POLD3
HCFC1
FOXK1
FOXK2
ASXL2
CBX6
RING1
PHC2
BAP1
UBAP2L
KDM1B
PHC3
CBX4
ASXL1
PHC1
CBX8
PCGF6
TERF2IP
TP53BP1
TRIM25
MDC1
RNF168
RFC1
BARD1
NBN
BABAM1
EXO1
PRKDC
UIMC1
MUS81
FAM175A
PALB2
SLX4
EME1

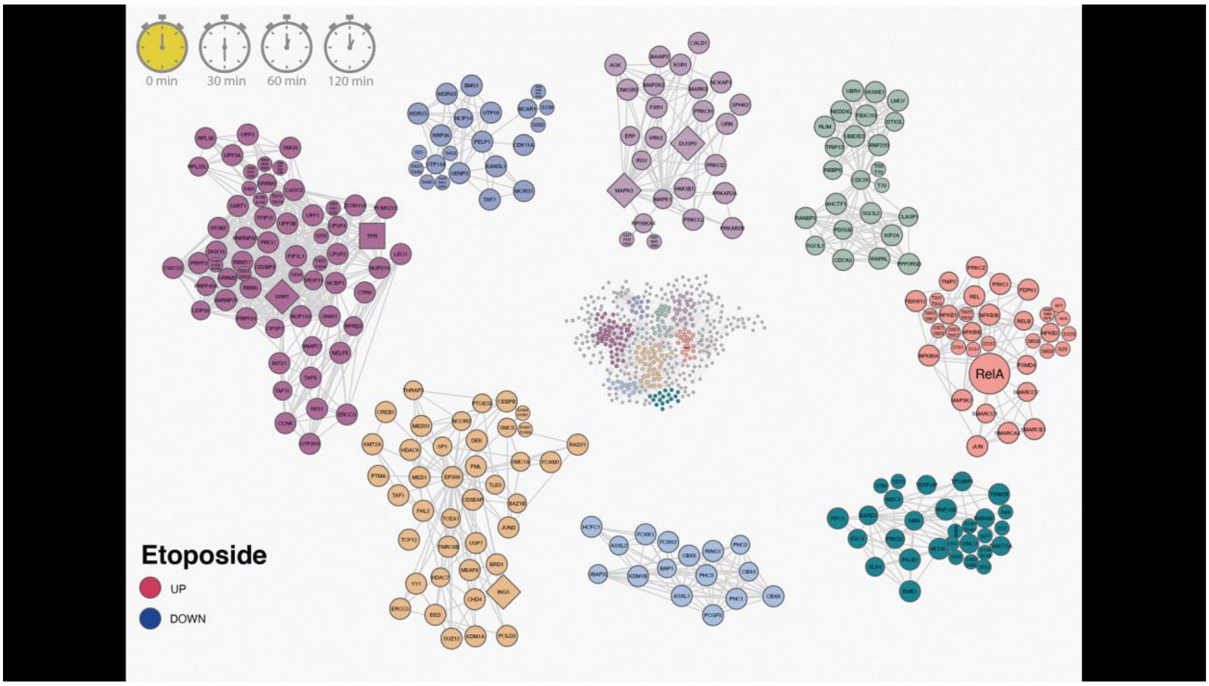
0 min
30 min
60 min
120 min
RelA
Etoposide
UP
DOWN

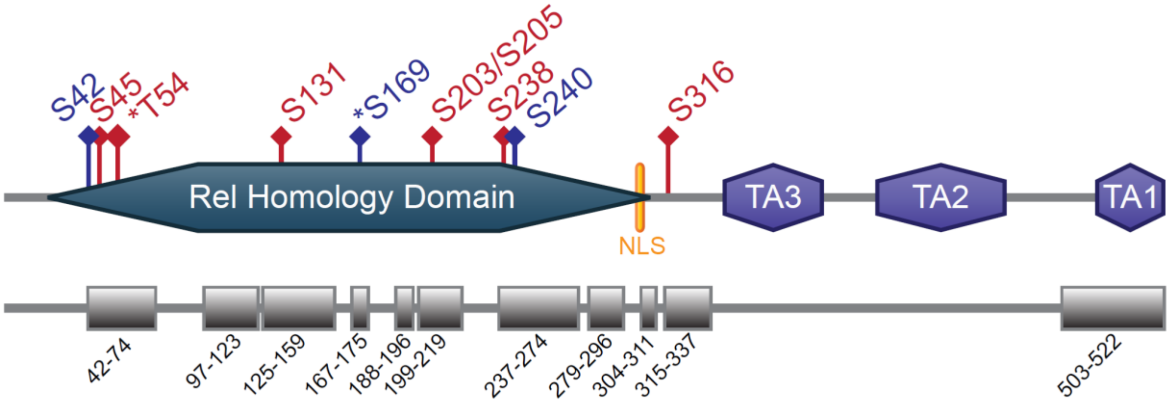
S42
S45
*T54
S131
*S169
S203/S205
S238
S240
S316
Rel Homology Domain
NLS
TA3
TA2
TA1
42-74
97-123
125-159
167-175
188-196
199-219
237-274
279-296
304-311
315-337
503-522
